# Surface Oxidation and Defect Formation Under Five Photoaging Regimes Enhance Cd^2+^ and Pb^2+^ Adsorption by Polyethylene and Polyvinyl Chloride Microplastics

**DOI:** 10.3390/molecules31183181

**Published:** 2026-09-10

**Authors:** Yichao Gong, Lu Liu, Yajing Guo, Pengyan Liu

**Affiliations:** 1 College of Chemical Engineering and Biotechnology, Xingtai University, Xingtai 054001, China; 2 State Key Laboratory of Environmental Chemistry and Ecotoxicology, Chinese Academy of Sciences, Research Center for Eco-Environmental Sciences, Beijing 100085, China; 3 School of Eco-Environment, Hebei University, Baoding 071000, China; hbu354340284@163.com (L.L.); hbupyliu@163.com (P.L.); 4 Environmental Protection Monitoring Center, SINOPEC Zhongyuan Oilfield Branch, Puyang 457001, China; 5 School of Clinical Medicine, Xingtai Medical College, Xingtai 054000, China

**Keywords:** microplastics, photoaging, polyethylene, polyvinyl chloride, surface oxidation, surface defects, metal ion adsorption

## Abstract

Photoaging alters polymer surface chemistry and can thereby regulate the interfacial binding of metal ions by microplastics. In this study, polyethylene (PE) and polyvinyl chloride (PVC) microplastics were exposed for three weeks to five regimes comprising UV-air, UV-ultrapure water, UV-simulated seawater, UV/H_2_O_2_, and UV/Cl. Changes in surface morphology, functional groups, crystallinity, hydrophobicity, and specific surface area were characterized before Cd^2+^ and Pb^2+^ adsorption. UV/Cl and UV/H_2_O_2_ induced the strongest transformations in both polymers, although the rate of aging decreased with exposure time. Photoaging generated surface cracks and defects, increased the accessible surface area, reduced hydrophobicity, and introduced hydroxyl, carbonyl, and carboxyl groups. These changes significantly enhanced Cd^2+^ and Pb^2+^ adsorption. Adsorption involved physical and chemical contributions, with chemical interactions predominating. FTIR, elemental mapping, and surface analyses were consistent with coordination and surface complexation at oxygen-containing sites, electrostatic attraction, and nonspecific physical retention on roughened surfaces and within defects. Variations in crystallinity and surface area further modulated adsorption. The results show that aging medium and polymer structure jointly determine the extent of surface oxidation and defect formation, establishing a structure–property relationship between photoaging-induced surface evolution and the enhanced metal-binding capacity of PE and PVC microplastics.

## 1. Introduction

Synthetic polymers are indispensable in modern manufacturing because of their low cost, durability, chemical resistance, and ease of processing. These same properties, however, allow discarded plastics to persist after their release into natural environments. Under ultraviolet irradiation, mechanical abrasion, temperature fluctuations, and chemical weathering, plastic products gradually lose their structural integrity and fragment into microplastics (MPs), generally defined as plastic particles smaller than 5 mm [1,2,3]. MPs can also enter aquatic and terrestrial environments through wastewater discharge, surface runoff, sewage sludge application, atmospheric deposition, and the fragmentation of discarded plastic products [4,5,6]. Their prolonged environmental residence permits further oxidation, chain scission, surface restructuring, and fragmentation, so their chemical and interfacial properties may differ substantially from those of the original polymers.

Polyethylene (PE), polypropylene, polyvinyl chloride (PVC), and polystyrene are among the most widely produced polymers and are common precursors of environmental MPs [7]. PE and PVC provide a useful comparison because their molecular structures and initial surface properties are markedly different. PE consists primarily of a nonpolar, saturated hydrocarbon backbone, whereas PVC contains polar C–Cl bonds and can undergo dehydrochlorination under photochemical or thermal stress. Both polymers are extensively used in films, packaging, pipes, construction products, and other long-service-life materials, increasing the likelihood of their release as plastic residues [8]. Following fragmentation, MPs may alter the physical and biological properties of surrounding media and interact with organisms and other environmental components [9,10,11]. Their small particle size, persistent polymer matrix, and progressively changing surface chemistry also enable them to associate with coexisting contaminants [11]. The behavior of PE and PVC microplastics is therefore determined not only by their original polymer structures but also by the chemical transformations and surface defects generated during aging.

Cadmium and lead are persistent metal contaminants that can coexist with MPs in natural and engineered environments [12]. They may be released through mining, industrial emissions, wastewater discharge, waste disposal, and the use of contaminated materials [13]. Their mobility and persistence depend on their partitioning among dissolved species, suspended particles, sediments, organic matter, and other solid phases [14]. This partitioning affects metal retention, transport, bioavailability, and potential transfer to organisms [15,16,17]. Microplastic surfaces may provide an additional interfacial phase for the adsorption of Cd^2+^ and Pb^2+^. Previous studies have related metal-ion adsorption by MPs to electrostatic attraction, coordination with polar functional groups, surface complexation, and nonspecific physical retention [18,19,20]. Association with MPs may decrease dissolved metal concentrations under some conditions, whereas mobile metal-loaded particles may promote contaminant redistribution between environmental compartments [21]. The contribution of MPs to metal partitioning consequently depends on polymer composition, surface chemistry, aging state, particle morphology, and solution conditions.

Metal-ion adsorption by MPs is governed by the chemical nature and accessibility of surface sites, together with surface morphology, specific surface area, crystallinity, hydrophobicity, and surface charge. Solution pH, ionic strength, dissolved salts, competing ions, and metal speciation further regulate surface-site ionization and the stability of adsorbed species [22,23]. More broadly, recent studies of polymer-based sorption systems show that adsorption capacity and uptake kinetics cannot be interpreted from surface area or porosity alone. Drzeżdżon et al. demonstrated that a waste-polyurethane-derived hybrid containing poly(allyl alcohol) and carbon nanotubes achieved rapid phenol removal only when accessible mesoporosity was balanced with chemically active polymer-derived binding sites; excessive polymer coverage could restrict pore accessibility and reduce sorption performance [10]. Nevertheless, many adsorption studies have used pristine commercial particles with relatively smooth and chemically unmodified surfaces. Such materials do not adequately represent MPs exposed to ultraviolet radiation, oxygen, water, salts, disinfectants, and reactive oxidizing species during environmental residence [24,25]. Aging can modify the structural characteristics that initially distinguish PE from PVC and can therefore change both the extent and dominant pathways of metal adsorption.

Photoaging initiates a series of reactions within the polymer matrix and at the particle interface. In PE, radical oxidation may proceed through hydroperoxide formation, chain scission, and the generation of hydroxyl, carbonyl, and carboxyl groups. In PVC, irradiation can promote dehydrochlorination and the formation of unsaturated sequences, followed by oxidative modification of the exposed surface. These reactions may cause embrittlement and cracking, alter crystallinity, increase surface roughness and specific surface area, and reduce hydrophobicity [26,27]. Surface oxidation introduces oxygen-donor groups that may participate in the coordination and surface complexation of Cd^2+^ and Pb^2+^. At the same time, cracks and other surface defects increase the accessibility of adsorption sites and provide additional regions for physical retention. The extent of oxidation and defect formation depends strongly on the aging medium because oxygen availability, water, dissolved salts, and oxidizing species influence radical reactions, mass transfer, and the stability of intermediate products. Irradiation in air, ultrapure water, saline water, or oxidant-containing solutions may therefore produce distinct surface chemistries and metal-binding properties.

Although aging-induced changes in microplastic adsorption have received increasing attention, the roles of polymer structure and aging medium have not been fully separated. Many studies have examined a single polymer, one aging treatment, or one adsorption endpoint. Direct comparisons of PE and PVC exposed for the same period to several contrasting photoaging regimes remain limited. Moreover, the relationships among surface oxidation, defect formation, crystallinity, hydrophobicity, specific surface area, and metal-ion adsorption have not been resolved systematically. Mechanistic interpretations based on a single analytical method may also be unreliable because changes in infrared band intensity or position do not independently establish a specific metal–polymer bond. A chemically consistent interpretation requires adsorption data to be evaluated together with spectroscopic evidence, elemental distributions, surface morphology, crystallinity, wettability, and accessible surface area.

In this study, PE and PVC microplastics were exposed for three weeks to five photoaging regimes involving ultraviolet irradiation in air, ultrapure water, simulated seawater, H_2_O_2_ solution, and chlorine solution. These treatments enabled the effects of atmospheric, aqueous, saline, and oxidizing media to be compared under controlled irradiation conditions. Changes in surface morphology, functional groups, crystallinity, hydrophobicity, and specific surface area were characterized, after which the adsorption of Cd^2+^ and Pb^2+^ by pristine and aged particles was evaluated. It was hypothesized that oxidative photoaging would increase the abundance and accessibility of oxygen-containing surface sites and generate structural defects, thereby enhancing metal adsorption, while the magnitude of these changes would depend on polymer structure and aging medium. The strongest surface transformations occurred under UV/H_2_O_2_ and UV/Cl conditions. The enhanced adsorption of Cd^2+^ and Pb^2+^ was most consistent with coordination and surface complexation at oxygen-containing sites, electrostatic attraction, increased accessible surface area, and physical retention at surface defects. By establishing how five photoaging regimes differently modify PE and PVC, this study clarifies the chemical and interfacial basis for their enhanced metal-binding capacity.

## 2. Results and Discussion

### 2.1. Characterization of MPs

#### 2.1.1. Effects of Aging Media on the Surface Chemical and Structural Evolution of MPs

**Figure 1:**
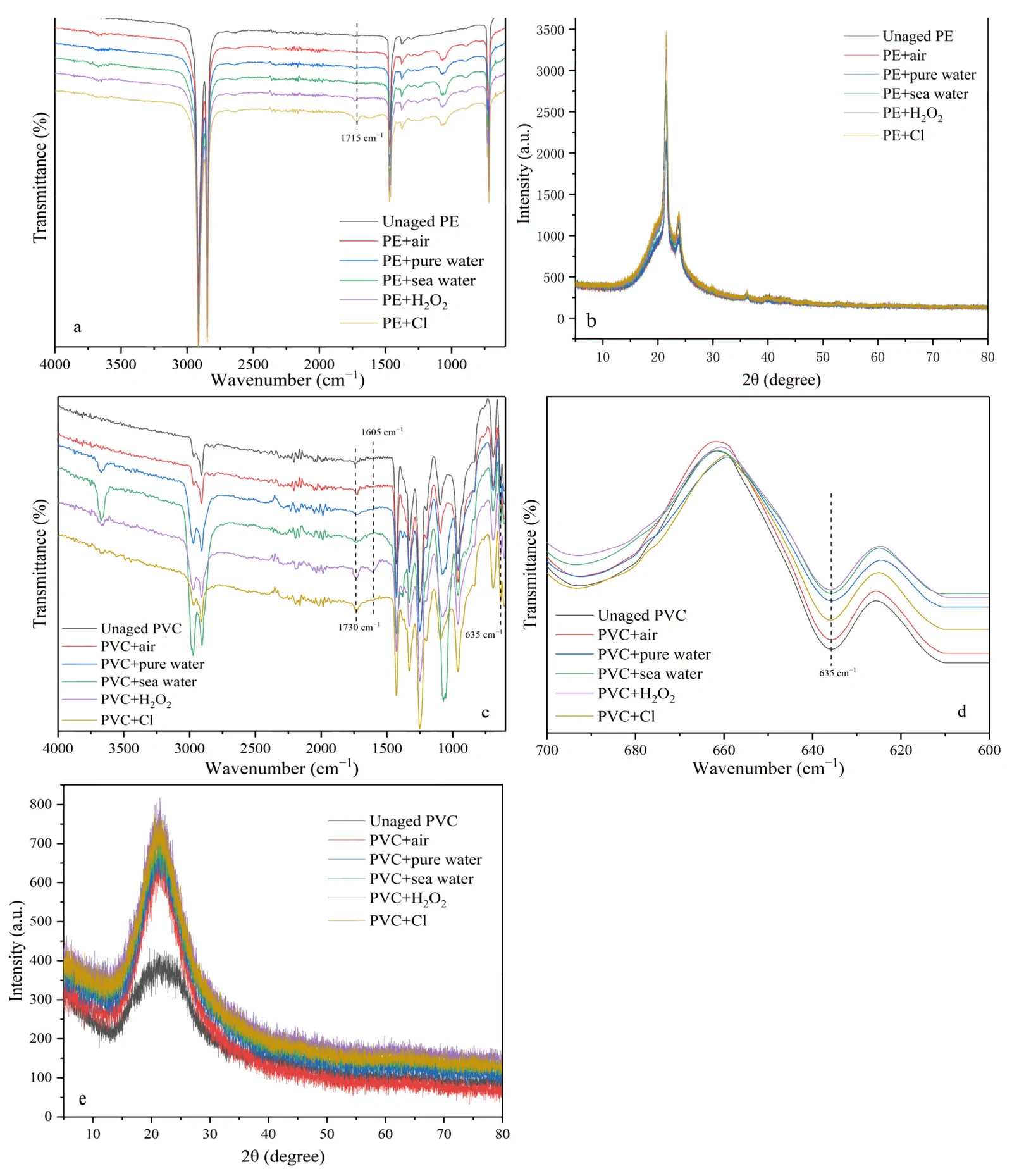
Characterization of PE and PVC under different aging conditions: (**a**) Infrared spectra of PE; (**b**) XRD patterns of PE; (**c**) Infrared spectra of PVC; (**d**) Magnified view of the C-Cl stretching vibration peak in PVC; (**e**) XRD patterns of PVC.

The surface chemical changes in PE and PVC after three weeks of exposure to the five photoaging regimes were examined using ATR-FTIR, as shown in Figure 1a,c,d. For PE, aging resulted in the appearance and progressive intensification of a carbonyl absorption band within 1800–1650 cm^−1^, with a prominent contribution near 1715 cm^−1^. This band is consistent with the formation of ketone, aldehyde, carboxylic acid, and related oxidation products during photooxidation. The principal C–H stretching and deformation bands of the PE backbone remained dominant, indicating that oxidation modified only a fraction of the chains within the ATR sampling region. For PVC, a carbonyl absorption band appeared within 1690–1740 cm^−1^, together with a band near 1605 cm^−1^ assigned to unsaturated C=C sequences. These changes are consistent with oxidative modification occurring concurrently with dehydrochlorination. The weak feature near 3700 cm^−1^ lies at the high-wavenumber edge of the O–H stretching region. It may contain contributions from isolated or weakly hydrogen-bonded hydroxyl groups, hydroperoxide-related species, and surface-adsorbed moisture [25]. However, residual atmospheric water vapor arising from differences between the background and sample scans may also produce weak features in this region [26]. Its intensity did not provide a consistent basis for ranking the aging treatments and was therefore not used as quantitative evidence of hydroxyl formation. The oxidation of PVC was evaluated primarily from the carbonyl, C=C, and C–Cl regions together with the complementary surface-characterization results. The absorption bands near 690–700 and 635 cm^−1^ belong to the characteristic C–Cl stretching region of PVC. PVC can exhibit several conformation-sensitive C–Cl stretching components, commonly located near 610, 635, and 690 cm^−1^. Variations in these bands may result from the loss of C–Cl groups during dehydrochlorination and from changes in local chain conformation or packing [24]. The observed decrease in the 635 cm^−1^ band and the variability of the band near 690–700 cm^−1^ are therefore consistent with dehydrochlorination and structural reorganization. They do not indicate the formation of new C–Cl bonds. The carbonyl index was used as a comparative measure of carbonyl accumulation rather than as a complete measure of polymer degradation [26]. For PE (Table 1), the CI values increased in the order PE-UV/air < PE-UV/ultrapure water < PE-UV/simulated seawater < PE-UV/H_2_O_2_ < PE-UV/Cl. For PVC (Table 1), the corresponding order was PVC-UV/air < PVC-UV/ultrapure water < PVC-UV/simulated seawater < PVC-UV/Cl < PVC-UV/H_2_O_2_. These results indicate that UV/Cl produced the greatest net carbonyl accumulation on PE, whereas UV/H_2_O_2_ produced the greatest carbonyl accumulation on PVC. The CI trends should not be interpreted independently as direct measurements of chain scission, cross-linking, or dehydrochlorination because the observed carbonyl content reflects the balance among product formation, further oxidation, surface erosion, and loss of low-molecular-weight products. The photoaging of PE can be described primarily by a radical autoxidation pathway [27]. UV excitation of pre-existing chromophores, hydroperoxides, or structural defects generates polymer macroradicals. Oxygen addition produces peroxy radicals, which abstract hydrogen from neighboring chains and form hydroperoxides. Subsequent hydroperoxide decomposition generates alkoxy radicals and other reactive intermediates. Alkoxy-radical β-scission and related reactions shorten the polymer chains and produce carbonyl, carboxyl, hydroxyl, and unsaturated end groups [26,27]. Recombination of polymer radicals may also result in branching or cross-linking.

**Scheme 1:**
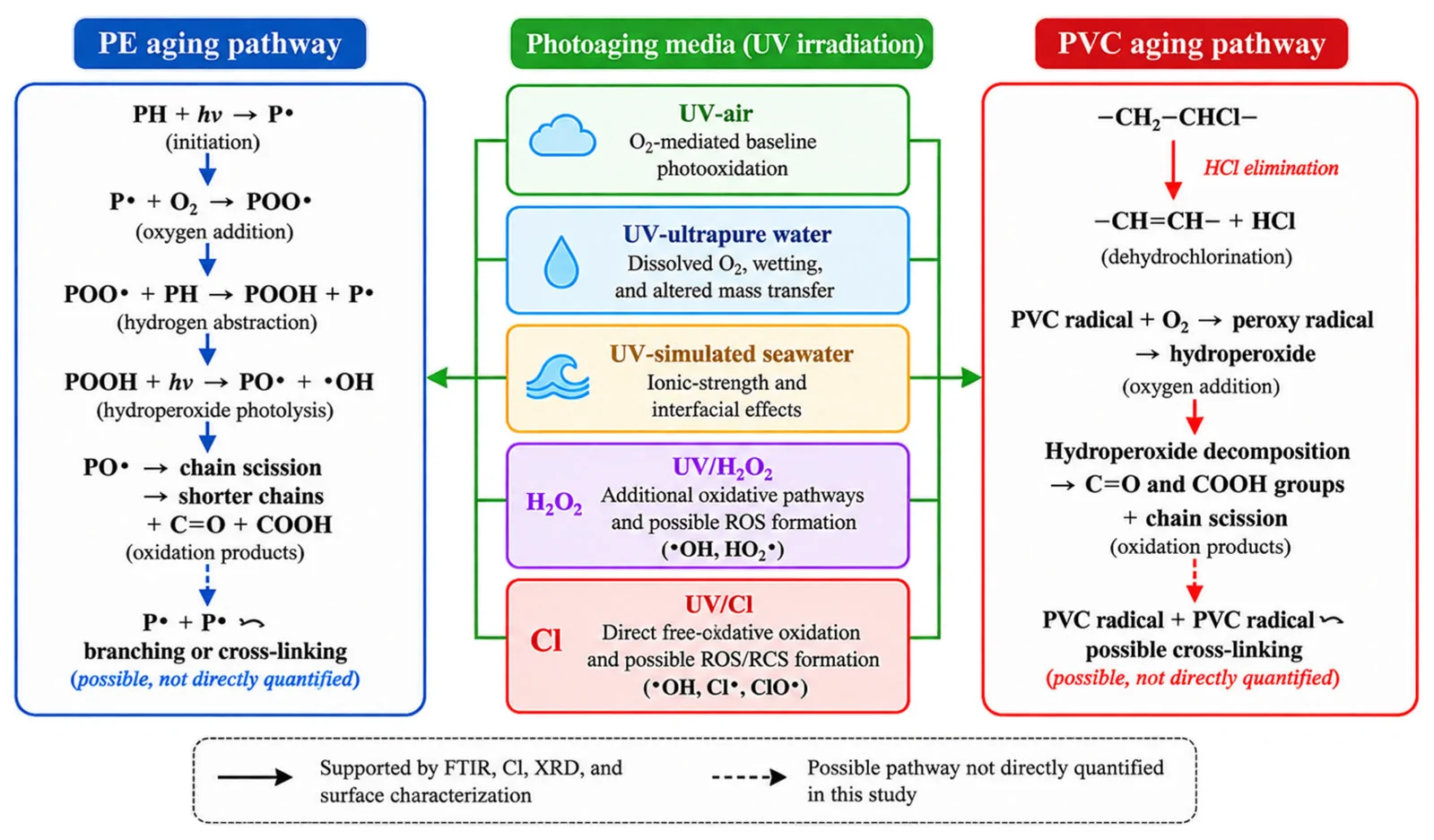
Proposed photoaging pathways of PE and PVC under the five aging regimes (ROS, reactive oxygen species; RCS, reactive chlorine species).

PVC follows a different initial pathway because of the presence of C–Cl bonds. UV exposure can initiate dehydrochlorination through elimination of HCl from adjacent carbon atoms, producing unsaturated sequences along the polymer backbone. The decrease in the C–Cl stretching bands and the appearance of the C=C band are consistent with this process. Polymer radicals and unsaturated segments can subsequently react with oxygen to form peroxy radicals and hydroperoxides. Decomposition of these intermediates generates carbonyl- and carboxyl-containing products and may cause chain scission and surface fragmentation. Radical recombination may also produce cross-linked structures, but the extent of this pathway was not quantified [24,27]. The spectral changes therefore support coupled dehydrochlorination and oxidation rather than direct substitution of chlorine by hydrogen or the formation of new C–Cl bonds. Differences among the five media arose from the combined effects of oxygen availability, oxidant chemistry, particle wetting, ionic composition, and mass transfer. UV-air represented baseline photooxidation driven by irradiation and atmospheric oxygen without an externally added oxidant. Ultrapure water altered particle wetting, heat transfer, and dissolved-oxygen transport. Simulated seawater additionally changed ionic strength, interfacial properties, and oxygen solubility, which may influence the persistence and accessibility of reactive intermediates. H_2_O_2_ provided an additional oxidation pathway and may undergo wavelength-dependent photochemical reactions that generate reactive oxygen species (ROS) Free chlorine can participate in direct oxidation and in wavelength- and pH-dependent photochemistry involving both reactive oxygen species and reactive chlorine species (RCS) [27]. The stronger response of PE under UV/Cl and of PVC under UV/H_2_O_2_ reflects the interaction between polymer-specific reactivity and the chemistry of the aging medium. Scheme 1 summarizes the proposed transformations of PE and PVC and the principal effects of the five aging media. Solid arrows indicate pathways supported by the FTIR, CI, XRD, and surface-characterization results, whereas dashed arrows denote possible radical recombination or cross-linking pathways that were not directly quantified.

The structural organization of PE and PVC after aging was further examined using XRD, as shown in Figure 1b,e. The XRD-derived apparent crystallinity was calculated by separating the integrated contributions of ordered domains from the broad amorphous scattering, as described in Section 3.2. The PE patterns contained a broad amorphous contribution centered at approximately 2θ = 19–20° and three resolved crystalline reflections. The two dominant reflections at approximately 2θ = 21.4° and 23.8° were assigned to the (110) and (200) planes, respectively, of orthorhombic PE, while the weaker reflection near 2θ = 36.1° was assigned to the (020) plane. The corresponding interplanar spacings were approximately 4.15, 3.74, and 2.49 Å, respectively. No additional resolved reflections or systematic peak shifts appeared after aging, indicating that the treatments did not produce a new crystalline polymorph of PE. The XRD-derived apparent crystallinity of PE increased from 34.81% in the unaged sample to 38.60%, 40.48%, 42.22%, 47.98%, and 49.48% after UV/air, UV/ultrapure water, UV/simulated seawater, UV/H_2_O_2_, and UV/Cl aging, respectively. The increase in the relative ordered contribution may result from preferential chain scission in less ordered regions, loss of degraded disordered material, and subsequent rearrangement or secondary crystallization of shortened chain segments. The treatment order was consistent with the carbonyl-index trend for PE. This correspondence indicates that the regimes producing greater surface oxidation also caused greater changes in the relative contribution of ordered domains, but it does not establish a direct causal relationship between carbonyl formation and crystallization.

All PVC samples were dominated by a broad diffuse halo centered approximately within 2θ = 21–23°, and no discrete Bragg reflections could be resolved reliably. Miller indices were therefore not assigned to the PVC halo. A crystallographic index would require the resolved ordered contribution to conform to an identifiable unit cell, which could not be established from the present powder patterns. No new sharp diffraction peaks appeared after aging, and the results do not indicate the formation of a new PVC crystalline polymorph. After identical background correction and profile fitting, the XRD-derived apparent crystallinity increased from 6.30% for unaged PVC to 16.70%, 26.50%, 38.26%, 56.82%, and 60.90% after UV/air, UV/ultrapure water, UV/simulated seawater, UV/Cl, and UV/H_2_O_2_ aging, respectively. Because the ordered contribution overlaps strongly with the broad amorphous scattering of PVC, these values are comparative and semiquantitative rather than absolute crystalline-phase fractions. The increase may reflect preferential degradation or removal of disordered chain segments and limited rearrangement of shortened chains into more ordered configurations. The relatively modest changes in the overall PVC ATR-FTIR profiles and the more pronounced XRD changes are not contradictory. ATR-FTIR primarily probes local bonding environments within the near-surface region, and its overall spectrum remains dominated by the PVC backbone even when a limited fraction of chains is oxidized or dehydrochlorinated. XRD is more sensitive to longer-range chain packing and to changes in the relative contributions of ordered and disordered domains. Chain rearrangement can therefore produce a substantial change in XRD-derived ordering without generating new chemical bonds sufficiently abundant to dominate the FTIR fingerprint. The combined results indicate that UV/H_2_O_2_ and UV/Cl caused the greatest surface oxidation, dehydrochlorination, and structural reorganization of PVC under the conditions examined.

#### 2.1.2. Time-Resolved Surface Chemical Evolution Under the Strongest Aging Regimes

**Figure 2:**
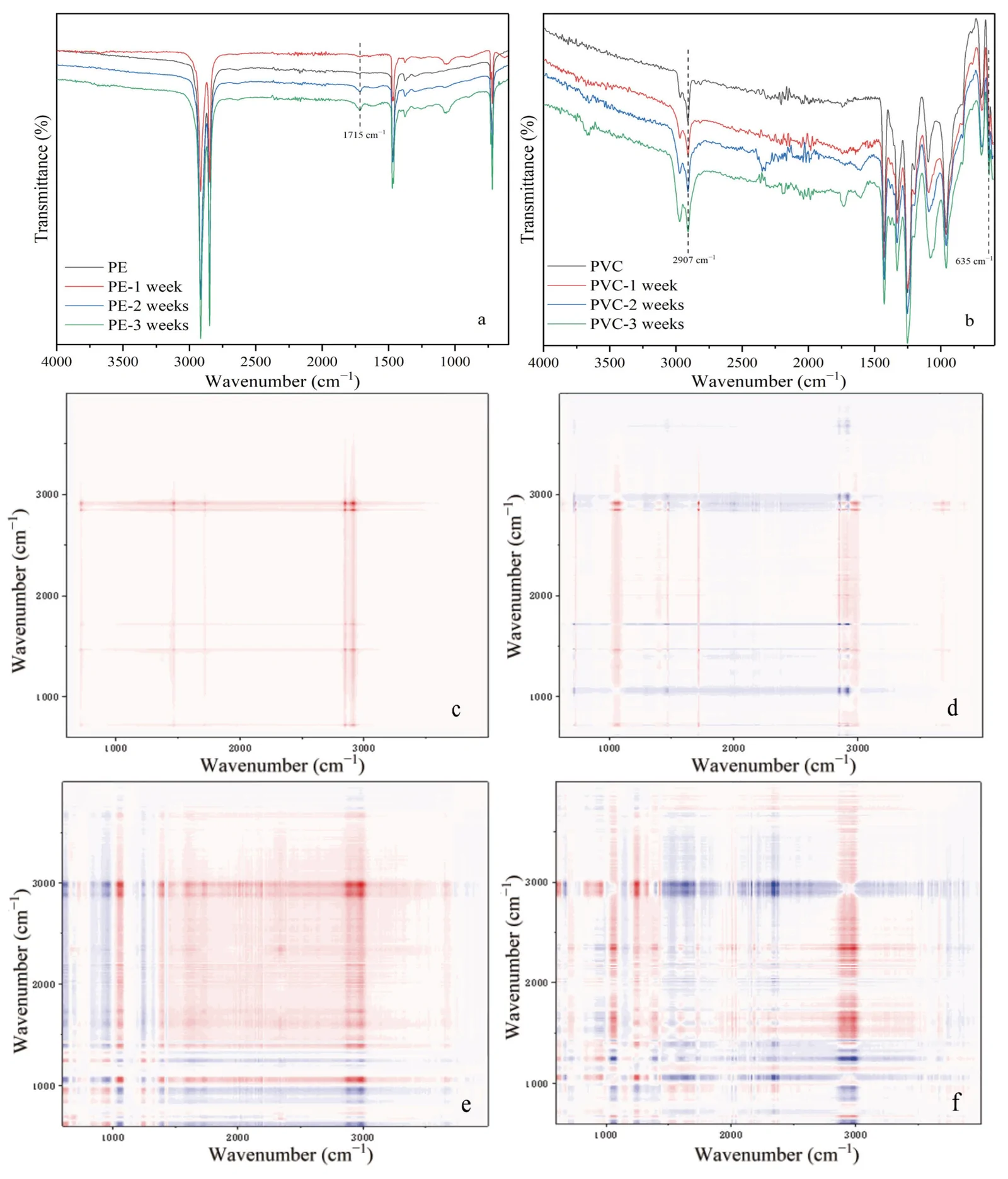
Infrared spectra and two-dimensional correlation spectra of PE and PVC under different aging conditions: (**a**) Infrared spectra of PE at various aging cycles under UV/Cl conditions; (**b**) Infrared spectra of PVC at various aging cycles under UV/H_2_O_2_ conditions; (**c**,**d**) Synchronous and asynchronous 2D correlation spectra of aged PE under UV/Cl conditions; (**e**,**f**) Synchronous and asynchronous 2D correlation spectra of aged PVC under UV/H_2_O_2_ conditions.

PE-UV/Cl and PVC-UV/H_2_O_2_ were selected for time-resolved analysis because they exhibited the most pronounced surface oxidation and XRD-derived structural reorganization within their respective polymer series after three weeks. The purpose of this analysis was to resolve spectral evolution under the strongest observed aging condition for each polymer rather than to establish a common temporal sequence for all five media, as shown in Figure 2a,b. The CI values for PE and PVC at various aging periods were calculated, as presented in Table 2. For PE, the carbonyl band near 1715 cm^−1^ and the corresponding CI increased with exposure time, confirming progressive accumulation of carbonyl-containing products [24]. The large increase during the initial period followed by a smaller incremental change is consistent with rapid reaction at readily accessible surface sites, followed by increasing limitations associated with oxygen or oxidant transport, loss of susceptible groups, and accumulation of oxidation products. For PVC, the band near 2907 cm^−1^ was assigned to C–H stretching, while the band at 635 cm^−1^ was assigned to C–Cl stretching [26,27]. The progressive decrease in the C–Cl band, together with the appearance of C=C and carbonyl bands, is consistent with coupled dehydrochlorination and oxidation [25,27]. The spectral changes do not indicate that hydrogen atoms directly replaced chlorine atoms. Dehydrochlorination involves elimination of HCl from adjacent carbon atoms, producing unsaturated chain segments. The increase in the carbonyl band and CI indicates subsequent oxidative modification of these chains.

To further elucidate the sequence of functional group changes during the aging process and the aging mechanisms of PE and PVC, 2D-COS analysis was performed based on the one-dimensional FTIR spectral data. The results are shown in Figure 2c–f. For PE under UV/Cl conditions, the synchronous spectrum contained autopeaks at 1471, 1715, 2849, and 2915 cm^−1^. Application of Noda’s rules gave the following order of spectral responses: 1715 cm^−1^ > 1471 cm^−1^ > 2849 cm^−1^ > 2915 cm^−1^. The earlier response of the carbonyl band relative to the principal C–H bands is consistent with rapid accumulation of oxidation products at the particle surface, followed by more extensive perturbation of the hydrocarbon backbone [24]. This order describes the sequence of band-intensity changes and should not be interpreted as direct proof that each elementary chemical reaction occurred in the same sequence. The 2D-COS data for PE under UV/Cl conditions are summarized in Table 3. According to the data, the order of changes in infrared absorption intensity with extended aging is as follows: 1715 cm^−1^ (C=O) > 1471 cm^−1^ (C-H) > 2849 cm^−1^ (C-H) > 2915 cm^−1^ (C-H). This indicates that under UV/Cl conditions, carbonyl groups are first generated in PE, followed by changes in the crystalline structure as aging progresses. The 2D-COS data for PVC under UV/H_2_O_2_ conditions are summarized in Table 3. For PVC under UV/H_2_O_2_ conditions, autopeaks were observed at 635, 1244, 1395, 1650, 1724, and 2946 cm^−1^, corresponding to C–Cl, CH_2_, C–H, C=C, C=O, and C–H-related vibrations, respectively. The spectral-response order was 2946 cm^−1^ > 1395 cm^−1^ > 1724 cm^−1^ > 635 cm^−1^ > 1650 cm^−1^ > 1244 cm^−1^. This sequence indicates that changes in the C–H environment preceded the major carbonyl, C–Cl, C=C, and CH_2_ spectral responses [27]. The result is consistent with coupled hydrogen abstraction, oxidation, and dehydrochlorination during PVC aging [25]. However, the 2D-COS results do not independently establish the exact sequence of bond cleavage, HCl elimination, oxygen addition, or radical recombination.

**Figure 3:**
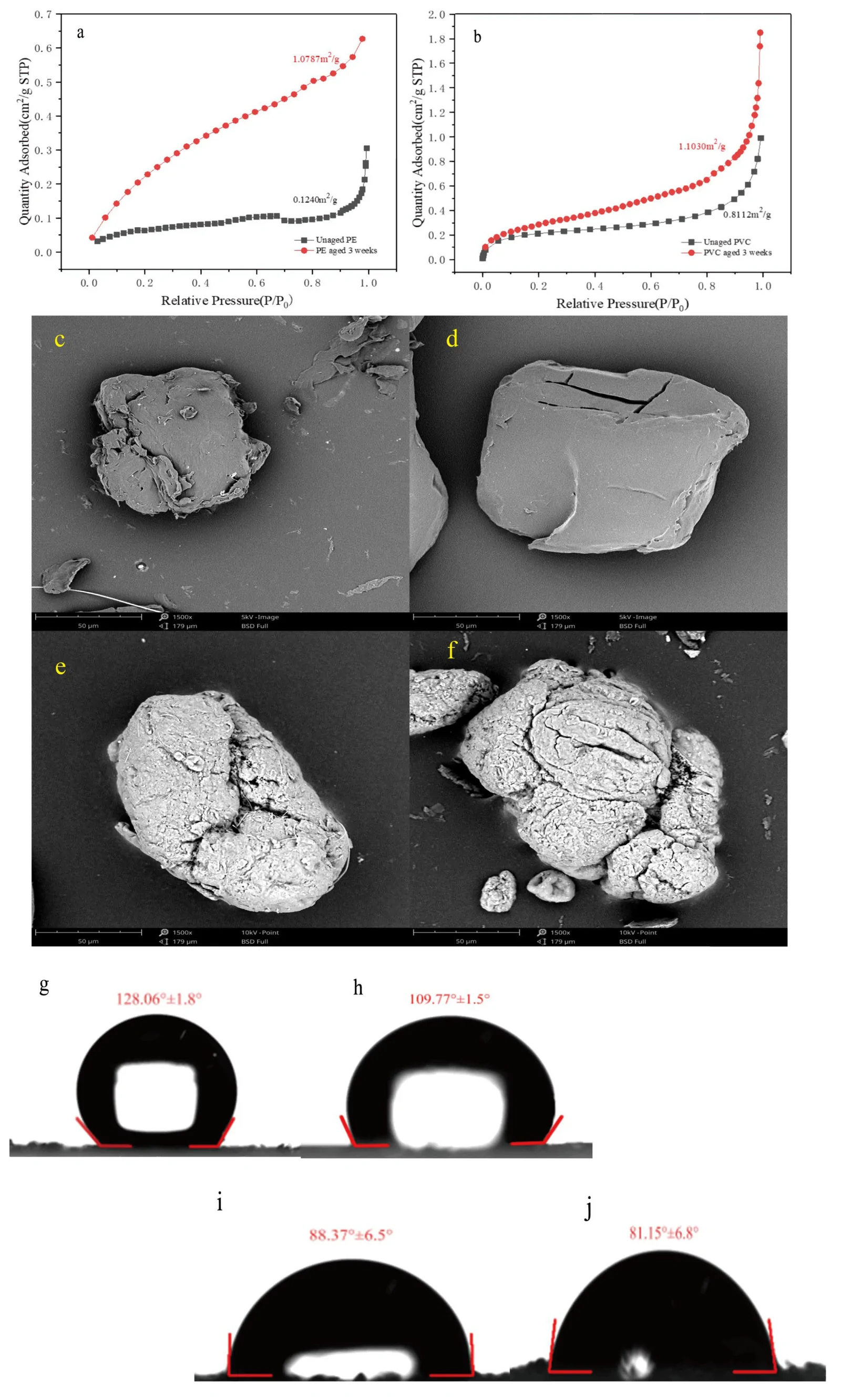
Characterization of unaged and aged PE and PVC: (**a**,**b**) BET adsorption isotherms of aged and unaged PE and PVC, respectively; (**c**,**d**) SEM images of unaged and aged PE; (**e**,**f**) SEM images of unaged and aged PVC; (**g**,**h**) Water contact angle measurements of unaged and aged PE; (**i**,**j**) Water contact angle measurements of unaged and aged PVC.

In addition to functional groups, the surface properties of aged PE and PVC were also studied, as shown in Figure 3. After 3 weeks of UV/Cl aging, the BET specific surface area of PE increased from 0.1240 to 1.0787 m^2^ g^−1^. The aged value was 8.70 times that of unaged PE, corresponding to a 769.9% increase (Figure 3a). This increase is attributed to surface cracking, fragmentation, and the formation of newly accessible surfaces during aging. Although cracks may facilitate the transport of oxygen or dissolved oxidants to freshly exposed regions, the present measurements do not demonstrate uniform oxidation of the particle interior. Similarly, after 3 weeks of UV/H_2_O_2_ aging, the BET specific surface area of PVC increased from 0.8112 to 1.1030 m^2^ g^−1^. The aged value was 1.36 times that of unaged PVC, corresponding to a 36.0% increase (Figure 3b). This indicates that UV/ H_2_O_2_ oxidation disrupted the chemical bonds in the PVC structure, causing large particles to fragment into smaller ones. The SEM images in Figure 3c–f were acquired to examine local surface morphology. The surface of unaged PE was comparatively smooth, whereas PE-UV/Cl exhibited pronounced cracks and local surface damage. Unaged PVC also showed a relatively compact surface, while PVC-UV/H_2_O_2_ exhibited greater roughness, cracking, and irregular surface features. These observations support aging-induced surface deterioration and defect formation. The combined BET and SEM results indicate that photoaging increased the accessible surface area primarily in association with surface roughening, crack formation, and the development of surface defects. These changes may expose additional adsorption regions and facilitate access to oxygen-containing binding sites. Thus, aged PVC exhibits relatively larger specific surface areas and smaller particle sizes. The hydrophobicity of PE and PVC also changed after aging. Contact angle measurements showed that the unaged PE had a contact angle of 128.06 ± 1.8° (Figure 3g), which decreased to 109.77 ± 1.5° after aging (Figure 3h). This reduction is because unaged PE primarily consists of nonpolar or weakly polar elements like C and H, making its surface resistant to wetting and highly hydrophobic. As aging progressed, hydrophilic groups such as carbonyl (C=O) were introduced, making the surface more susceptible to wetting and liquid penetration, thereby reducing hydrophobicity. Similarly, the hydrophilicity of PVC increased after aging, with the water contact angle decreasing from 88.37 ± 6.5° (Figure 3i) to 81.15 ± 6.8° (Figure 3j). This is attributed to the introduction of hydrophilic groups such as C=O (ketones and aldehydes), which enhanced wetting and penetration of liquids into the PVC surface, reducing its hydrophobicity.

### 2.2. Adsorption Kinetics and Isotherms

Detailed adsorption experiments were conducted using unaged PE, unaged PVC, PE-UV/Cl after three weeks, and PVC-UV/H_2_O_2_ after three weeks. The two aged samples were selected because they exhibited the most pronounced surface and structural transformations within their respective polymer series during the initial five-regime screening. In the following sections, “PE-UV/Cl” and “PVC-UV/H_2_O_2_” refer specifically to these three-week-aged samples. The adsorption experiments were designed to compare pristine polymers with their most strongly transformed endpoints.

#### 2.2.1. Adsorption Kinetics

**Figure 4:**
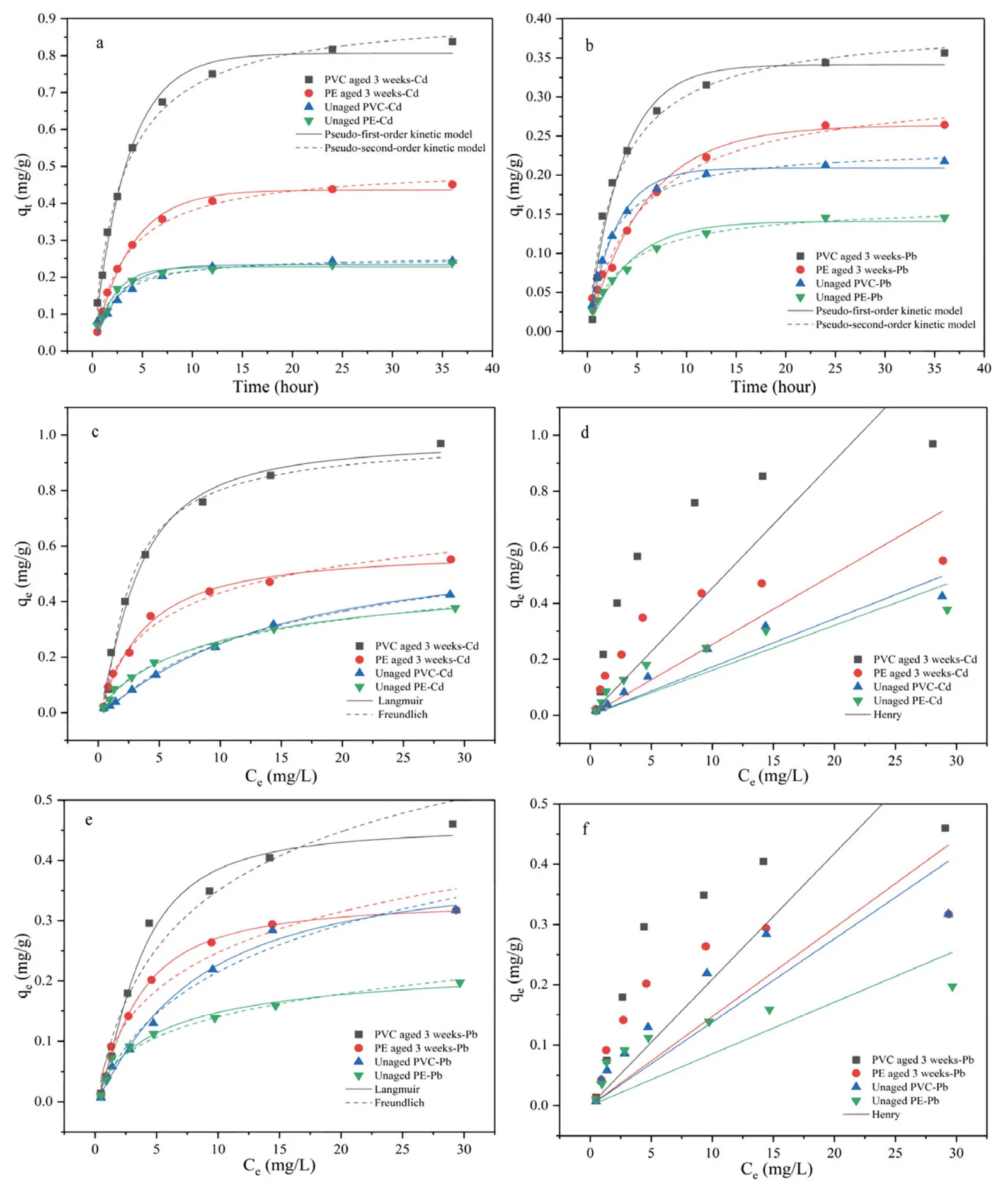
Adsorption kinetics and isotherm models of Cd^2+^ and Pb^2+^ by PE and PVC before and after aging: Adsorption kinetics of Cd^2+^ and Pb^2+^ by aged and non-aged PE and PVC (**a**,**b**); Langmuir, Freundlich and Henry models for Cd^2+^ adsorption by PE and PVC before and after aging (**c**,**d**); Langmuir, Freundlich and Henry models for Pb^2+^ adsorption by PE and PVC before and after aging (**e**,**f**).

Figure 4a presents the adsorption kinetics curves of Cd^2+^ on unaged and aged PE and PVC. As shown in the figure, the adsorption of Cd^2+^ on all MPs reached equilibrium within 36 h, with equilibrium concentrations of 0.2389 mg/g for unaged PE, 0.4508 mg/g for aged PE, 0.2443 mg/g for unaged PVC, and 0.8379 mg/g for aged PVC. The adsorption kinetics process was fitted using pseudo-first-order and pseudo-second-order kinetic models, and the fitted kinetic parameters are listed in Table 4. From the results in the table, the average correlation coefficient *R*^2^ for the pseudo-second-order kinetic model was 0.9853, while that for the pseudo-first-order model was 0.9704. Therefore, the pseudo-second-order kinetic model better describes the adsorption process of Cd^2+^ on both unaged and aged PE and PVC, indicating that the adsorption process is primarily governed by chemical adsorption.

Figure 4b shows the adsorption kinetics curves of Pb^2+^ on unaged and aged PE and PVC. From the figure, it can be observed that the adsorption of Pb^2+^ on all MPs reached equilibrium within 36 h, with equilibrium concentrations of 0.1458 mg/g for unaged PE, 0.2641 mg/g for aged PE, 0.2175 mg/g for unaged PVC, and 0.3565 mg/g for aged PVC. The adsorption kinetics process was fitted using pseudo-first-order and pseudo-second-order kinetic models, and the corresponding kinetic parameters are presented in Table 4. From the results in the table, the average correlation coefficient *R*^2^ for the pseudo-second-order kinetic model was 0.9870, whereas the average *R*^2^ for the pseudo-first-order model was 0.9802. This indicates that the pseudo-second-order kinetic model better describes the adsorption process of Pb^2+^ on both unaged and aged PE and PVC, suggesting that the adsorption process is primarily dominated by chemical adsorption.

The factors influencing the adsorption of heavy metals by unaged and aged PE and PVC can be categorized into two main aspects. On one hand, the surface adsorption properties of MPs, including hydrophobic interactions and specific surface area, play a crucial role. The adsorption capacity order derived from the adsorption kinetics experiments was unaged PE < unaged PVC < aged PE < aged PVC. This sequence aligns with their specific surface area order: unaged PE (0.1240 m^2^/g) < unaged PVC (0.8112 m^2^/g) < aged PE (1.0787 m^2^/g) < aged PVC (1.1030 m^2^/g). This indicates a strong correlation between the adsorption capacity of MPs and their specific surface area. Characterization of the aged MPs showed that they had smaller particle sizes, larger specific surface areas, greater hydrophilicity, higher crystallinity, and more oxygen-containing functional groups. These factors contribute to the increased adsorption capacity of aged PE and PVC. Specifically, in the Cd^2+^ adsorption experiments, the adsorption capacity of PE increased by 88.8%, while that of PVC increased by 2.43 times. In the Pb^2+^ adsorption experiments, the adsorption capacity of PE increased by 81.2%, and that of PVC increased by 63.9%. On the other hand, the type of heavy metal also affects the adsorption process on MPs. The experiments revealed that the adsorption capacity of the tested MPs for Cd^2+^ was greater than for Pb^2+^. This is attributed to the different atomic numbers and surface valence states of Cd and Pb, leading to variations in surface potential. Consequently, the interaction forces between the charged or polar regions of the MPs and the heavy metal ions differ, thereby influencing the adsorption behavior.

#### 2.2.2. Adsorption Isotherms

Figure 4c–e, and f illustrate the adsorption isotherms of Cd^2+^ and Pb^2+^ on unaged and aged PE and PVC. The adsorption capacity follows the order: aged PVC > aged PE > unaged PVC > unaged PE. The results indicate that the aging process significantly alters the surface hydrophobicity, crystallinity, surface oxidation (evaluated by the carbonyl index, CI), and specific surface area of MPs, thereby influencing their interaction with heavy metals. Consequently, the adsorption capacity of aged PE and PVC for heavy metals is notably enhanced. The adsorption-isotherm experiments were designed to characterize the concentration-dependent binding behavior and to estimate the Langmuir-derived apparent maximum adsorption capacity q_m_ rather than to reproduce natural aquatic exposure conditions. The initial metal concentrations of 0.5–30 mg L^−1^ and the microplastic loading of 2.0 g L^−1^ were intentionally selected to provide a sufficiently broad equilibrium range and to approach the saturation region required for estimation of q_m_. Accordingly, the fitted q_m_ values should be regarded as laboratory-derived capacity parameters under the specified pH, temperature, solution composition, and solid-to-liquid ratio. The equilibrium adsorption modeling were fitted using the Langmuir, Freundlich, and Henry models, and the thermodynamic parameters are presented in Table 5. According to the results, the Langmuir model provided the best fit for the adsorption of Cd^2+^ and Pb^2+^, with average correlation coefficients (R^2^) of 0.9937 and 0.9865, respectively. The Freundlich model yielded average R^2^ values of 0.9928 and 0.9676, while the Henry model had lower average R^2^ values of 0.8546 and 0.8217. These findings suggest that the Langmuir model is better suited to describe the adsorption of heavy metals onto unaged and aged MPs, indicating that Cd^2+^ and Pb^2+^ adsorption is primarily monolayer adsorption, involving both physical and chemical adsorption, with chemical adsorption being dominant. The Langmuir model describes monolayer homogeneous adsorption in a macroscopic sense. Despite the irregular shapes and heterogeneous surfaces of MPs observed under SEM, the system appears homogeneous on a macroscopic level, and the solution remains well-mixed during stirring [28]. Hence, the adsorption of heavy metals onto MPs can be represented by the Langmuir isotherm.

The adsorption process proceeds in three stages. In the initial stage, heavy metal ions diffuse from the bulk solution to the water film formed on the surface of MPs due to hydration. The ions overcome the resistance of the liquid film and cross it to reach the surface of MPs, a process dominated by van der Waals forces and electrostatic interactions, where physical adsorption plays a key role. In the second stage, heavy metal ions on the outer surface of MPs diffuse through pores to inner surface adsorption sites. Here, valence forces are generated through electron sharing or exchange between the ions and active sites on the MPs, constituting a chemical adsorption process that requires energy consumption. The third stage involves the adsorption reaching a quasi-equilibrium state. The partition coefficients (K_d_) for Cd^2+^ and Pb^2+^ on MPs range from 0.0161–0.0454 L/g and 0.0086–0.0209 L/g, respectively. The agreement of the equilibrium data with the Langmuir equation does not imply that adsorption capacity should scale with BET specific surface area. The fitted capacity reflects the number and affinity of sites available to the metal ions under the aqueous experimental conditions, whereas BET analysis quantifies nitrogen-accessible surface area under dry conditions. The nonproportional changes summarized in Table S3 further indicate that surface chemistry and site accessibility contributed substantially to the observed adsorption behavior.

### 2.3. Effects of the External Factors

#### 2.3.1. Effects of pH

**Figure 5:**
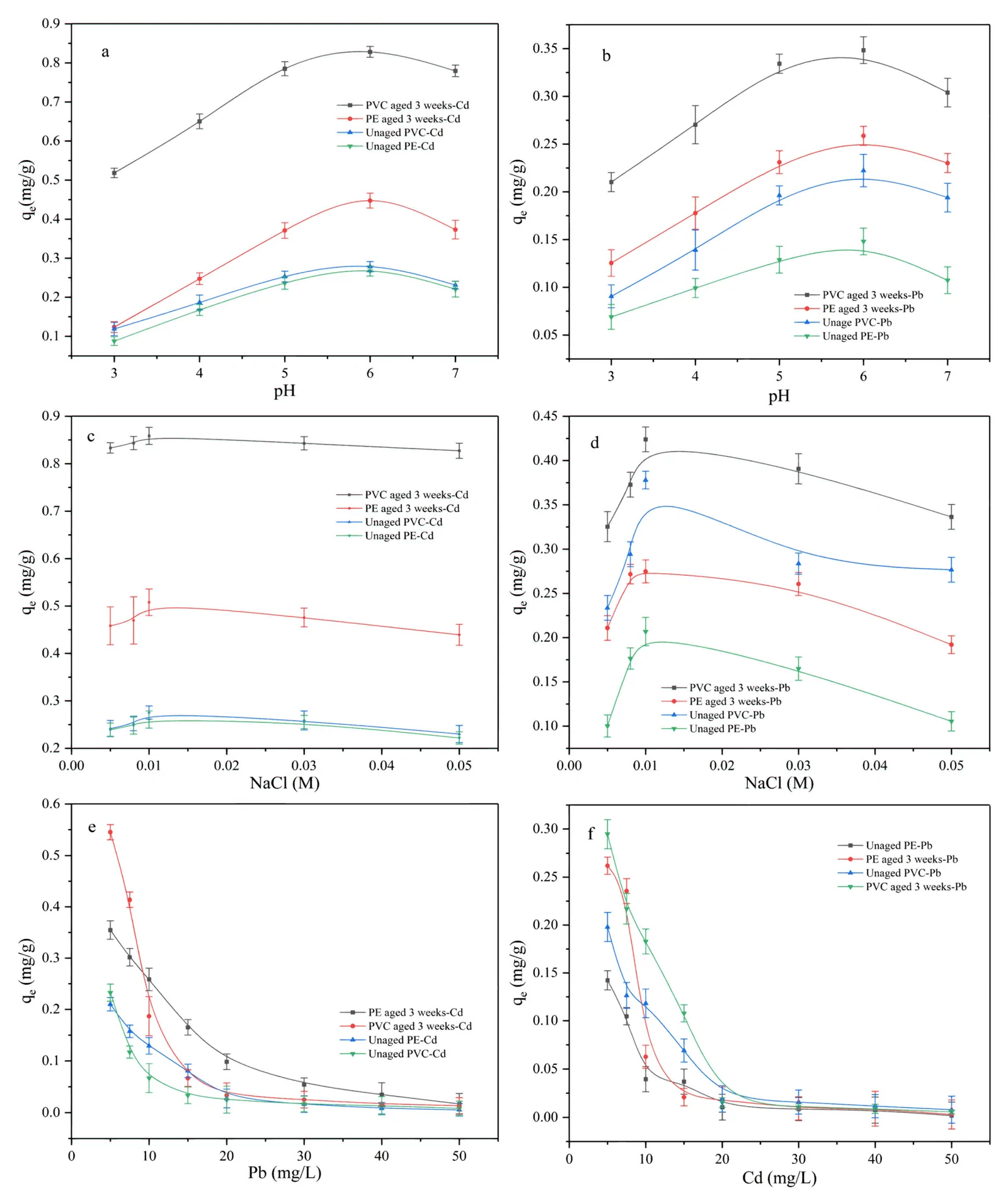
Adsorption of Cd^2+^ and Pb^2+^ by PE and PVC before and after aging under different conditions: (**a**,**b**) effects of pH on Cd^2+^ and Pb^2+^ adsorption; (**c**,**d**) effects of salinity on Cd^2+^ and Pb^2+^ adsorption; (**e**) competitive adsorption of Pb^2+^ with varying Pb^2+^ concentrations; (**f**) competitive adsorption of Cd^2+^ with varying Cd^2+^ concentrations.

Since pH affects both the surface charge of adsorbents and the speciation of metal ions, it is widely recognized as a crucial factor influencing the adsorption performance of MPs in aquatic environments [29]. The results are shown in Figure 5a,b. As the solution pH increased, the adsorption capacity of MPs for Cd^2+^ and Pb^2+^ initially increased and then decreased, reaching a maximum at pH 6.0. The increase in adsorption capacity can be attributed to enhanced anionic surface characteristics within this pH range, indicating that electrostatic interactions play a significant role in the adsorption of heavy metals by MPs. Furthermore, the electrostatic interactions between aged MPs and Cd^2+^ and Pb^2+^ are enhanced [25]. The point of zero charge (pH_pzc_) of PE and PVC is approximately 5.8. When pH > pH_pzc_, MPs carrying a net negative charge are more favorable for heavy metal adsorption [30]. However, if the solution pH continues to increase, charge repulsion occurs, leading to a reduction in adsorption capacity. Additionally, at higher pH levels, Cd^2+^ and Pb^2+^ form precipitates such as Cd(OH)^+^, Cd(OH)_2_, Pb(OH)^+^, Pb(OH)_2_, and Pb(OH)_3_^−^ [31]. These precipitates compete for the active sites on the surface of MPs, resulting in a decrease in their adsorption capacity for Cd^2+^ and Pb^2+^ [32]. Based on the dissociation constants (Ksp) of Cd and Pb, the pH at which 10 mg/L Cd^2+^ and Pb^2+^ solutions begin to form precipitates can be calculated as 6.7 and 6.2, respectively, which aligns closely with the findings of this study. Therefore, the adsorption interactions between unaged and aged PE and PVC with Cd^2+^ and Pb^2+^ are primarily governed by ion exchange and electrostatic interactions [33].

#### 2.3.2. Effect of Salinity

Salinity can influence the adsorption behavior between MPs and metals through several mechanisms. Figure 5c,d illustrate the effect of different salinities (0.005, 0.008, 0.01, 0.03, and 0.05 M) on the adsorption of Cd^2+^ and Pb^2+^. At 0.01 M NaCl, the maximum adsorption capacities of different MPs for Cd^2+^ were 0.8586 mg/g (aged PVC), 0.5079 mg/g (aged PE), 0.2754 mg/g (unaged PVC), and 0.2606 mg/g (unaged PE); for Pb^2+^, the maximum adsorption capacities were 0.4239 mg/g (aged PVC), 0.2748 mg/g (aged PE), 0.3779 mg/g (unaged PVC), and 0.2068 mg/g (unaged PE). At 0.05 M NaCl, the maximum adsorption capacities of different MPs for Cd^2+^ were 0.8272 mg/g (aged PVC), 0.4391 mg/g (aged PE), 0.2301 mg/g (unaged PVC), and 0.2219 mg/g (unaged PE); for Pb^2+^, the maximum adsorption capacities were 0.3363 mg/g (aged PVC), 0.1919 mg/g (aged PE), 0.2768 mg/g (unaged PVC), and 0.1054 mg/g (unaged PE). These results indicate that the adsorption of Cd^2+^ and Pb^2+^ by both aged and unaged PE and PVC increases at lower salinity levels but decreases at higher salinity levels. This phenomenon can be attributed to two primary factors: First, the Na^+^ in NaCl competes with Cd^2+^ and Pb^2+^ for adsorption sites, while Cl^−^ inhibits Cd^2+^ and Pb^2+^ through the formation of complexes such as CdCl^+^, CdCl_2_, CdCl_3_^−^, CdOHCl, PbCl^+^, and PbCl_2_. Second, increased salinity causes aggregation of MPs, reducing the number of available adsorption sites. Additionally, salinity introduces electrostatic shielding effects on the metals, impairing their electrostatic adsorption behavior [31].

#### 2.3.3. Effect of Competitive Adsorption

Given that multiple heavy metals coexist in the environment, the competitive adsorption of different heavy metals was investigated. Figure 5e,f show the differences in the adsorption capacities of Cd^2+^ and Pb^2+^ by aged and unaged PE and PVC in mixed metal solutions. The results indicate that the adsorption capacity in the mixed solution was lower than that in single-metal solutions, suggesting the occurrence of competitive adsorption. As the concentration of Pb^2+^ or Cd^2+^ ions increased, the adsorption of Cd^2+^ or Pb^2+^ by MPs decreased. Furthermore, at the same ion concentration (10 mg/L), the adsorption of Cd^2+^ by MPs was higher than that of Pb^2+^. This phenomenon can be attributed to two primary reasons: First, Cd^2+^ and Pb^2+^ share the same adsorption sites on MPs [32]. The binding of MPs with Pb^2+^ or Cd^2+^ (5–50 mg/L) inhibits the adsorption of the other metal ion. Second, the molar mass of Pb (207.2 g/mol) is significantly higher than that of Cd (112.4 g/mol). Consequently, at a given mass concentration of Cd and Pb (mg/L), the molar concentration of Pb (mmol/L) is always lower than that of Cd.

### 2.4. Adsorption Mechanism

**Figure 6:**
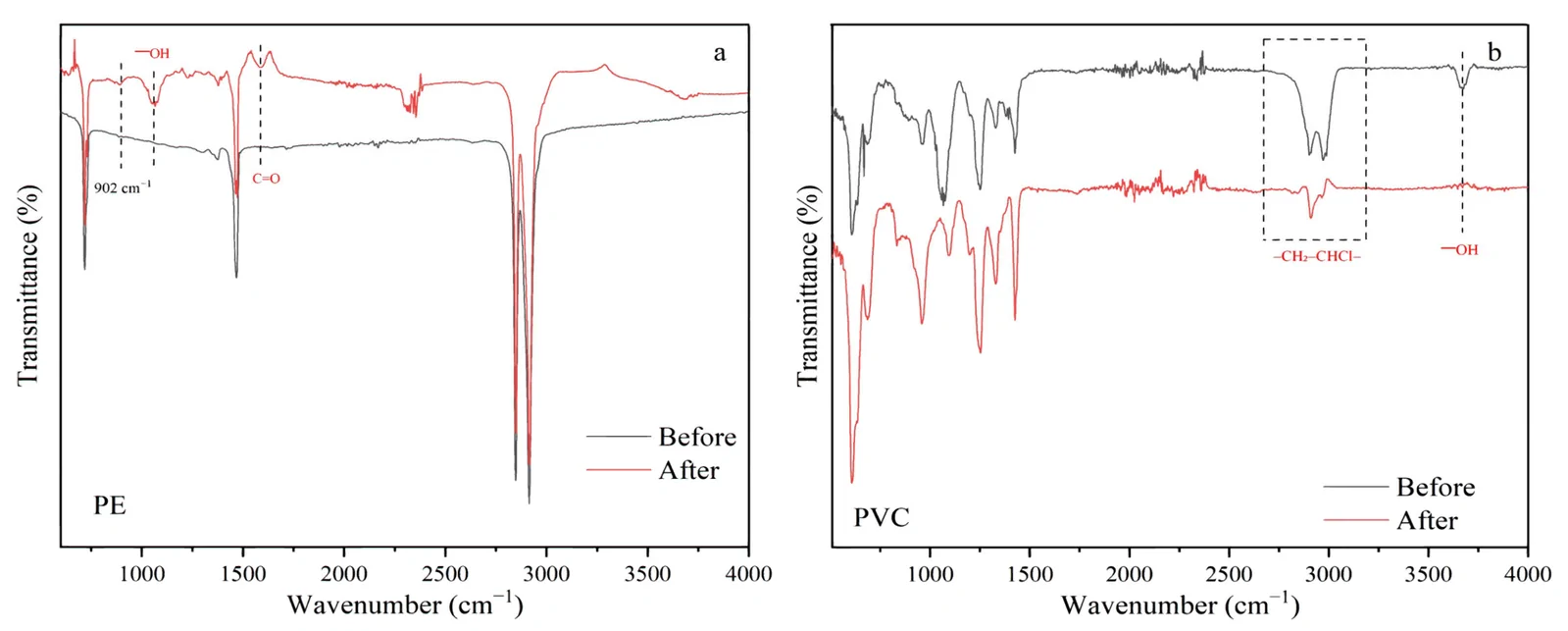
FTIR spectra of PE (**a**) and PVC (**b**) before and after the adsorption of heavy metals.

Figure 6a,b compare the FTIR spectra of PE and PVC before and after Cd^2+^ and Pb^2+^ adsorption. The principal absorption bands associated with the polymer backbones remained largely unchanged after adsorption, whereas moderate variations in band intensity and position occurred in regions associated with oxygen-containing functional groups. These variations indicate that adsorption altered the local vibrational environment of existing surface groups rather than producing new functional groups or substantially modifying the polymer backbones. Accordingly, the appearance or increased prominence of O–H and C=O bands after adsorption should not be interpreted as the formation of new hydroxyl or carbonyl groups. Instead, these changes suggest the involvement of oxygen-containing groups that had already formed during photoaging. The intensity of an infrared-active vibration is proportional to the square of the derivative of the dipole moment with respect to the corresponding normal coordinate. Adsorption-induced polarization or partial electron donation can therefore change the transition dipole of a surface group and alter its normalized infrared intensity. Uniform changes across the entire spectrum were not assigned to adsorption because ATR intensity can also vary with particle packing and contact with the ATR crystal [34].

For PE, variations in the O–H and carbonyl regions indicate that aging-derived polar groups participated in Cd^2+^ and Pb^2+^ adsorption. Carbonyl and carboxyl groups can provide oxygen-donor sites for metal coordination and surface complexation. When carboxyl groups are deprotonated, the resulting negatively charged sites may also promote electrostatic attraction toward Cd^2+^ and Pb^2+^. Changes in the O–H region may reflect modifications in the hydration environment or interactions among surface functional groups, but they do not demonstrate direct hydrogen bonding between the metal ions and PE. The absorption band near 902 cm^−1^ falls within the characteristic region of vinyl C–H wagging in PE and is associated with unsaturated structures formed during polymer photooxidation rather than with a newly formed metal–polymer bond [35,36]. Because PE has a predominantly saturated aliphatic structure and contains neither aromatic rings nor an extended conjugated π-electron system, π–π interactions were excluded from the proposed adsorption mechanism. The increased surface roughness, cracking, and specific surface area caused by aging may additionally promote nonspecific physical adsorption and retention at surface defects.

For PVC, changes in the O–H and C=O regions similarly indicate the participation of oxygen-containing groups introduced during photoaging. The observed variations in the C–H bands and in the 700–1000 cm^−1^ region reflect changes in the local environment of polymer skeletal and C–H deformation modes and should not be assigned directly to adsorbed Cd^2+^ or Pb^2+^. Although the covalent C–Cl bonds contribute to the intrinsic polarity of PVC, their presence alone does not establish negatively charged adsorption sites or demonstrate direct bonding between chlorine and the metal ions. Photoaging of PVC involves both dehydrochlorination and oxidation. Elimination of HCl can generate isolated or conjugated C=C sequences, whereas oxidation introduces hydroxyl, carbonyl, and carboxyl groups. The oxygen-containing groups provide the most directly supported binding sites for Cd^2+^ and Pb^2+^ through coordination, surface complexation, and electrostatic attraction. The presence of unsaturated sequences does not support a π–π stacking mechanism for Cd^2+^ or Pb^2+^ adsorption. π–π stacking requires two interacting π-electron systems, whereas Cd^2+^ and Pb^2+^ are metal cations rather than π-containing adsorbates. A cation–π interaction is a distinct mechanism and may, in principle, occur between a metal cation and a sufficiently electron-rich and accessible π system. However, the present FTIR data establish only the formation of C=C-containing structures and do not determine their conjugation length, electron density, abundance, or surface accessibility. The available spectroscopic and elemental data also do not identify these unsaturated sequences as metal-binding sites. A cation–π contribution was therefore not assigned as a mechanism supported by the present study, although a minor contribution cannot be excluded solely on theoretical grounds.

The elemental maps in Figure S1 confirm the presence and spatial distribution of Cd and Pb on the surfaces of PE and PVC after adsorption, while the corresponding elemental compositions are presented in Table S2. The lower relative Cl signal and the appearance or increase in O in aged PVC are consistent with dehydrochlorination and oxidative surface modification. These changes do not indicate that C–H bonds replaced C–Cl bonds. Moreover, because the elemental percentages obtained by energy-dispersive X-ray spectroscopy are normalized, decreases in the relative proportions of C and Cl may partly reflect the appearance of oxygen and adsorbed metals. The higher relative Cd and Pb signals detected on aged MPs are consistent with the batch adsorption results and support the conclusion that aging increased the number or accessibility of metal-binding sites. However, elemental mapping confirms the presence of the adsorbed metals but does not identify their chemical state or establish a specific bonding mechanism.

Taken together, the spectroscopic, elemental, surface, and adsorption results indicate that the enhanced Cd^2+^ and Pb^2+^ adsorption by PE-UV/Cl and PVC-UV/H_2_O_2_ was controlled by the abundance, chemical character, ionization state, and aqueous accessibility of oxygen-containing surface groups. Coordination and surface complexation at these sites involved adsorption-induced polarization and electron-density redistribution, while electrostatic attraction contributed at negatively charged surface groups. Surface cracking, roughening, and defect formation increased nitrogen-accessible and interfacial area and provided additional regions for nonspecific physical retention. However, the adsorption capacities did not increase in direct proportion to BET specific surface area, demonstrating that geometrical surface enlargement alone did not determine metal binding. π–π stacking was excluded because the adsorbed metal ions did not provide a second π-electron system. Although a cation–π interaction is conceptually possible between a metal cation and an electron-rich π domain, the present data did not establish sufficiently conjugated and accessible π sites on aged PVC or identify such sites as being perturbed during adsorption. Cation–π binding was therefore not included among the experimentally supported mechanisms. Direct metal–polymer hydrogen bonding and direct coordination through C–Cl groups were likewise not supported. The available evidence most consistently supports coordination and surface complexation at oxygen-donor groups, electrostatic attraction, and a secondary contribution from nonspecific physical retention.

## 3. Materials and Methods

### 3.1. Materials

PE and PVC were purchased from the Dingsu Plastic Business Department (Dongguan, China), with a particle size of 80 mesh. Ethanol (C_2_H_5_OH, chromatographic grade, purity ≥ 99.9%) and concentrated nitric acid (HNO_3_, analytical grade, concentration 65–68%) were obtained from Kemiou Chemical Reagent Co., Ltd. (Tianjin, China). Hydrogen peroxide (H_2_O_2_, 30%, analytical grade, purity ≥ 99%), sodium chloride (NaCl, analytical grade, purity ≥ 99%), and magnesium chloride hexahydrate (MgCl_2_·6H_2_O, analytical grade, purity ≥ 99%) were purchased from Aladdin Bio-Chem Technology (Shanghai, China). Sodium hypochlorite solution (NaClO, analytical grade, purity ≥ 99%), sodium sulfate decahydrate (Na_2_SO_4_·10H_2_O, analytical grade, purity ≥ 99%), and sodium hydroxide (NaOH, analytical grade, purity ≥ 98%) were procured from Damao Chemical Reagent Factory (Tianjin, China). Cadmium standard solution (matrix: HNO_3_, concentration 1000 mg/L) and lead standard solution (matrix: HNO_3_, concentration 1000 mg/L) were obtained from the National Institute of Metrology, Certification and Accreditation (Beijing, China). Ultrapure water (prepared using the PURELAB Classic system, ELGA, High Wycombe, UK) was used for all experiments.

### 3.2. Characterization of MPs

The crystal phase structure and crystallinity of the MPs were analyzed using an X-ray diffractometer (XRD, D8 Advance, Bruker, Fällanden, Switzerland) equipped with a CuKα radiation source. The operating conditions included a voltage of 40 kV, a current of 40 mA, a scanning speed of 10°/min, and a scanning range of 5–80°. The XRD profiles were analyzed over the same 2θ range using an identical background-correction and profile-fitting procedure. After background subtraction, each profile was separated into diffraction contributions assigned to ordered domains and a broad amorphous halo by nonlinear least-squares fitting. The surface hydrophobicity of the MPs was measured using a contact angle goniometer (JC2000DM; Powereach, Beijing, China). During the test, ultrapure water droplets of approximately 6 μL were vertically dispensed onto the solid surface using a microsyringe at a fixed distance. The measurements were conducted at room temperature. The BET specific surface area and pore size distribution of the MPs were determined using a fully automatic surface area and porosity analyzer (ASAP2460; Micromeritics, Norcross, GA, USA) through N_2_ adsorption–desorption. Before the test, the MPs samples were degassed at 80 °C, followed by low-temperature adsorption at −196 °C. The surface morphology of the MPs was observed using a scanning electron microscope (SEM, JSM-7500, JEOL, Tokyo, Japan) at an accelerating voltage of 10 kV. SEM was used to compare local surface morphology, including cracking, roughening, and defect formation. The chemical composition of the MPs was identified using an attenuated total reflection Fourier transform infrared spectrometer (ATR-FTIR, TENSOR 27, Bruker, Switzerland) with a wavelength scanning range of 500–4000 cm^−1^ and a resolution of 2 cm^−1^. Because the penetration depth of the evanescent field is limited to the micrometre scale and varies with the ATR crystal, wavenumber, incidence angle, and refractive indices of the crystal and sample, the ATR-FTIR results were not used to determine the depth of the modified layer. All FTIR spectra were subjected to the same baseline-correction and integration procedure. For PE, the carbonyl index was calculated as the ratio of the integrated carbonyl-band area near 1715 cm^−1^ to the integrated reference-band area near 1470 cm^−1^. For PVC, the carbonyl index was calculated as the ratio of the integrated carbonyl-band area at 1690–1740 cm^−1^ to the integrated C–H reference region at 1450–1550 cm^−1^. Identical integration ranges were applied to all samples. The elemental composition of the MPs was analyzed using a scanning electron microscope equipped with an energy-dispersive spectrometer (Phenom ProX, Phenom-Scientific, Shanghai, China).

### 3.3. Aging Methods for MPs

**Figure 7:**
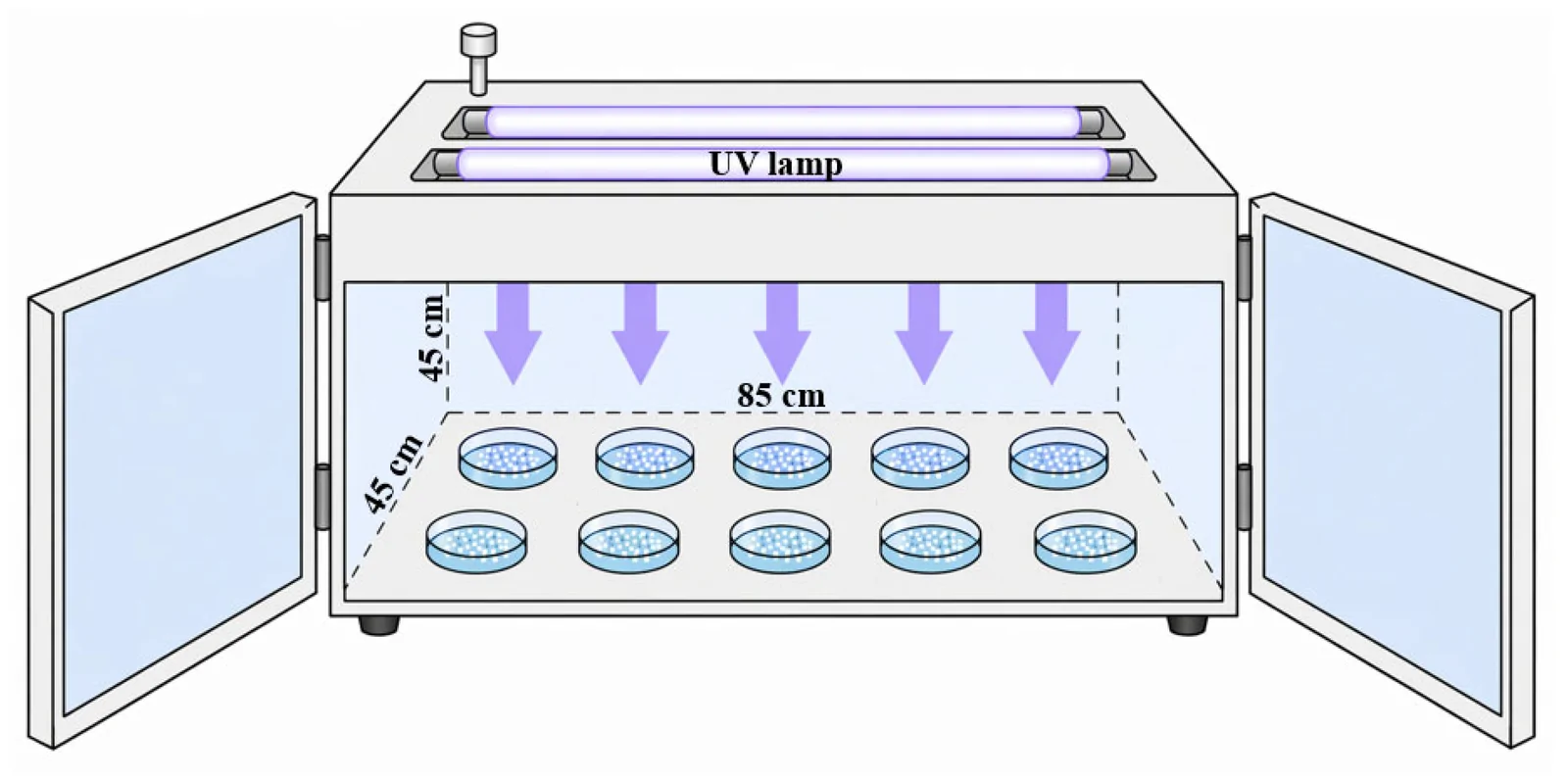
Aging chamber.

Accurately weigh 20.0 g of two types of MPs (PE and PVC) into separate 50 mL beakers. Fill the beakers with ethanol to submerge the MPs, and soak for 12 h. After soaking, wash thoroughly and immerse in ultrapure water for 24 h. Once the soaking process is complete, clean and dry the MPs for subsequent use. Aging experiments were conducted at room temperature in a custom aging chamber equipped with two UVA-340 fluorescent lamps (rated power: 20 W per lamp; Q-Lab, Westlake, OH, USA). The lamps had a peak emission wavelength of approximately 340 nm, consistent with the characteristic spectral output of UVA-340 lamps. The vertical distance between the lamp plane and the sample surface was maintained at 45 cm throughout the experiment. The UV aging setup and lamp-to-sample geometry are shown in Figure 7. The samples consisted of irregular 80-mesh particles rather than planar films; therefore, a uniform sample thickness and a single treatment depth could not be defined. Next, weigh 1.5 g of MPs into separate 50 mL beakers and sequentially add 20 mL of simulated environmental solutions (excluding the UV/air group). These solutions were replenished periodically. The aging period was three weeks, with samples collected weekly, washed, dried, and stored for future analysis. The simulated environmental solutions included ultrapure water, simulated seawater, hydrogen peroxide, and sodium hypochlorite solution. The simulated seawater (hereafter denoted as “sea water”) was prepared by accurately weighing 24.7 g of NaCl, 3.0 g of MgCl_2_·6H_2_O, and 3.97 g of Na_2_SO_4_·10H_2_O, dissolving them in ultrapure water, and making up the volume to 1 L. The solution was sealed and stored for later use. The resulting aged MPs were labeled as MPs-UV/air, MPs-UV/pure water, MPs-UV/sea water, MPs-UV/H_2_O_2_, and MPs-UV/Cl. Each group of samples was tested in triplicate.

### 3.4. Adsorption Experiments

In all adsorption experiments, the aged PE sample was PE exposed to UV/Cl for three weeks, and the aged PVC sample was PVC exposed to UV/H_2_O_2_ for three weeks. The four adsorbent groups were therefore unaged PE, PE-UV/Cl, unaged PVC, and PVC-UV/H_2_O_2_. These sample identities were used consistently in the kinetic, isotherm, pH, salinity, competitive adsorption, FTIR, and elemental-mapping experiments.

#### 3.4.1. Adsorption Kinetics

A 20.0 mg portion of each adsorbent, including unaged PE, PE-UV/Cl after three weeks, unaged PVC, and PVC-UV/H_2_O_2_ after three weeks, was placed in a separate 30 mL glass centrifuge tube. Separate adsorption experiments were conducted for Cd^2+^ and Pb^2+^. For each metal, 10 mL of a 10 mg L^−1^ solution adjusted to pH 6.0 was added to the corresponding tubes. Blank controls containing the same metal solution but no microplastics were prepared under identical conditions. The tubes were tightly capped and agitated in a thermostatic water-bath shaker at 150 rpm and 25 °C in the dark for 36 h. Samples were collected after 0.5, 1, 1.5, 2.5, 4, 7, 12, 24, and 36 h. At each designated sampling time, a 5 mL aliquot of the reaction solution was withdrawn using a syringe and passed through a 0.45 μm aqueous membrane filter. The residual dissolved Cd^2+^ or Pb^2+^ concentration was determined using a flame atomic absorption spectrophotometer (WFX-120B, BFRL, Ningbo, China). The metal concentration measured in the corresponding blank control was used to correct for concentration changes unrelated to adsorption. The adsorption capacities at time (t) and at equilibrium were calculated from the blank-corrected concentration differences according to Equations (1) and (2), respectively. All adsorption treatments and blank controls were performed in triplicate.

#### 3.4.2. Adsorption Isotherms

Solutions with initial concentrations of 0.5, 1, 1.5, 3, 5, 10, 15, and 30 mg/L of mixed heavy metal ions (Cd^2+^ and Pb^2+^, pH = 6.0) were prepared. Accurately weigh 20 mg of unaged MPs (PE, PVC) and aged MPs (PE, PVC) into separate 30 mL glass centrifuge tubes, and set up a blank control group. Add 10 mL of the prepared heavy metal ion solutions at the eight different concentrations to the unaged group, aged group, and blank group. All samples were shaken in the dark at 25 °C and 150 rpm for 36 h in a constant temperature shaker. After the reaction, extract 5 mL of the solution with a syringe and filter it through a 0.45 μm aqueous filter head. The concentrations of heavy metals in the solutions were analyzed using a flame atomic absorption spectrophotometer. The differences in heavy metal concentrations between the unaged and aged groups and the blank group were calculated. The adsorption capacity of MPs for heavy metals was determined using the differential method. Each experiment was conducted in triplicate.

#### 3.4.3. Influence Factor Experiment

To investigate the effect of pH on the adsorption properties of unaged and aged MPs, five pH gradients (3.0, 4.0, 5.0, 6.0, and 7.0) were set, considering the hydrolysis of Cd^2+^ and Pb^2+^ and their competitive adsorption with H^+^. The pH of the solutions was adjusted using 0.1 mol/L NaOH or 0.1 mol/L HNO_3_, and the concentration of Cd^2+^ and Pb^2+^ in the mixed solution was maintained at 10 mg/L. Precisely 20 mg of unaged MPs (PE, PVC) and aged MPs (PE, PVC) were weighed and placed in 30 mL glass centrifuge tubes. Then, 10 mL of heavy metal solutions with different pH values were added to each set of centrifuge tubes. All samples were incubated in a thermostatic shaker (25 °C, 150 rpm) for 36 h in the dark. Subsequently, 5 mL of the reaction solution was drawn using a syringe, filtered through a 0.45 μm aqueous filter, and analyzed for heavy metal concentrations using a flame atomic absorption spectrophotometer. The difference in heavy metal concentrations between the unaged and aged groups and the blank control group was calculated to determine the adsorption capacity of MPs using the differential method. All experiments were conducted in triplicate. Additionally, the effect of salinity on the adsorption capacity of unaged and aged MPs was studied. Solutions with salinities of 0.005, 0.008, 0.01, 0.03, and 0.05 M were prepared by diluting NaCl. The heavy metal concentration was maintained at 10 mg/L with a pH of 6.0. The same procedure was followed as described above. All experiments included blank controls, and samples were incubated in a thermostatic shaker (25 °C, 150 rpm) for 36 h in the dark. After incubation, 5 mL of the reaction solution was filtered through a 0.45 μm aqueous filter and analyzed for heavy metal concentrations using a flame atomic absorption spectrophotometer. The difference in heavy metal concentrations between the unaged and aged groups and the blank control group was calculated to determine the adsorption capacity of MPs using the differential method. All experiments were conducted in triplicate.

#### 3.4.4. Competitive Adsorption Experiment

In the experiment investigating the adsorption of Cd^2+^ by MPs, a Cd^2+^ solution (10 mg/L, pH = 6.0) containing Pb^2+^ (5–50 mg/L) was used as the adsorbate. Similarly, in the experiment investigating the adsorption of Pb^2+^ by MPs, a Pb^2+^ solution (10 mg/L, pH = 6.0) containing Cd^2+^ (5–50 mg/L) was used as the adsorbate. All other specific procedures were conducted as described above.

### 3.5. Adsorption Capacity of MPs

The adsorption capacity was used to indicate the adsorption capacity of adsorbent to sorbate:

(1) $$q_{t}=\frac{\left(C_{0}-C_{t}\right)\;V}{m}$$

(2) $$q_{e}=\frac{\left(C_{0}-C_{e}\right)\;V}{m}$$

where *q_t_* (μg/g) and *q_e_* (μg/g) are the amount of adsorption at t time and equilibrium, respectively; *C_t_* (μg/L) is the concentrations of adsorbate at the t time.

The adsorption kinetics and isotherm models used to analyze the experimental data are presented in Table S1.

## 4. Conclusions

This study systematically compared the photoaging behavior of polyethylene and polyvinyl chloride microplastics under five regimes involving UV irradiation in air, ultrapure water, simulated seawater, H_2_O_2_ solution, and chlorine solution. UV/Cl and UV/H_2_O_2_ induced the most pronounced surface oxidation and defect formation in both polymers, although the progression of aging slowed with increasing exposure time. Photoaging generated hydroxyl, carbonyl, and carboxyl groups, caused surface embrittlement and cracking, increased the specific surface area, reduced hydrophobicity, and altered crystallinity. These transformations significantly enhanced the adsorption of Cd^2+^ and Pb^2+^, demonstrating that both polymer structure and aging medium governed the extent of surface modification and metal binding.

The adsorption behavior reflected both physical and chemical contributions, with surface chemical interactions providing the principal contribution. The combined spectroscopic, elemental, and surface characterization results were consistent with coordination and surface complexation at oxygen-containing sites, together with electrostatic attraction at ionizable surface groups. Surface roughening, cracking, and defect formation increased the accessibility of adsorption sites and promoted nonspecific physical retention, while changes in crystallinity and specific surface area further modulated metal uptake. The enhanced adsorption therefore resulted from the combined effects of surface oxidation and morphological evolution rather than from a single interaction mechanism. These findings establish a structure–property relationship between photoaging-induced surface transformations and the increased metal-binding capacity of PE and PVC microplastics. Further studies using natural irradiation, chemically complex media, cross-sectional depth profiling, direct surface-speciation analyses, and desorption experiments are needed to determine the spatial extent of polymer modification and the stability and reversibility of Cd^2+^ and Pb^2+^ binding under environmentally relevant conditions.

## Figures and Tables

**Table 1:** Carbonyl index of PE and PVC under different aging conditions.

Aging Conditions	Peak Area	CI
PE-UV/air	0.0078	0.0033
PE-UV/pure water	0.1198	0.0500
PE-UV/sea water	0.1504	0.0628
PE-UV/H_2_O_2_	0.1702	0.0710
PE-UV/Cl	0.7090	0.2959
PVC-UV/air	0.0509	0.0342
PVC-UV/pure water	0.0670	0.0449
PVC-UV/sea water	0.0880	0.0590
PVC-UV/H_2_O_2_	0.1608	0.1078
PVC-UV/Cl	0.1486	0.0996

**Table 2:** Carbonyl index of PE and PVC under different aging cycles.

	Aging Cycle	Peak Area	CI
	1 week	0.0045	0.0019
PE	2 weeks	0.5820	0.2429
	3 weeks	0.7090	0.2959
	1 week	0.0140	0.0094
PVC	2 weeks	0.0173	0.0115
	3 weeks	0.1608	0.1078

**Table 3:** PE and PVC two-dimensional infrared spectrum data sheet.

	ν_1_ ν_2_ (cm^−1^)	Φ (ν_1,_ ν_2_)	Ψ (ν_1,_ ν_2_)
PE	1471, 1715	>0	<0
	1471, 2849	>0	>0
	1471, 2915	>0	>0
	1715, 2849	>0	>0
	1715, 2915	>0	>0
	2849, 2915	>0	>0
PVC	635, 1244	>0	>0
	635, 1395	<0	>0
	635, 1650	<0	<0
	635, 1724	<0	>0
	635, 2946	<0	>0
	1244, 1395	<0	>0
	1244, 1650	<0	>0
	1244, 1724	<0	>0
	1244, 2946	<0	>0
	1395, 1650	>0	>0
	1395, 1724	>0	>0
	1395, 2946	>0	<0
	1650, 1724	>0	<0
	1650, 2946	>0	<0
	1724, 2946	>0	<0

**Table 4:** Kinetic parameters for the sorption of Cd^2+^ and Pb^2+^ on PE and PVC microplastics before and after aging.

	Pseudo-First-Order Kinetic Model $q_{t}=q_{e}(1-e^{{-K}_{1}t})$			Pseudo-Second-Order Kinetic Model $q_{t}=\frac{q_{e}^{2}k_{2}t}{1+q_{e}k_{2}t}$		
Cd^2+^	*K*_1_ (h^−1^)	*q_e_* (mg/g)	*R* ^2^	*K*_2_ (h^−1^)	*q_e_* (mg/g)	*R* ^2^
Unaged PE	0.4976	0.2275	0.9737	2.5689	0.2530	0.9820
Aged PE	0.2710	0.4359	0.9953	0.6260	0.5018	0.9950
Unaged PVC	0.3886	0.2331	0.9205	2.0166	0.2585	0.9682
Aged PVC	0.2928	0.8063	0.9919	0.3763	0.9215	0.9961
Pb^2+^	*K*_1_ (h^−1^)	*q_e_* (mg/g)	*R* ^2^	*K*_2_ (h^−1^)	*q_e_* (mg/g)	*R* ^2^
Unaged PE	0.2365	0.1409	0.9683	1.7513	0.1620	0.9912
Aged PE	0.1698	0.2634	0.9850	0.5819	0.3146	0.9874
Unaged PVC	0.3513	0.2092	0.9920	1.8267	0.2361	0.9957
Aged PVC	0.2870	0.3413	0.9756	0.8319	0.3936	0.9736

**Table 5:** Adsorption isotherms simulation parameters of Cd^2+^ and Pb^2+^ on PE and PVC microplastics before and after aging.

	Langmuir Model $q_{e}=q_{m}\frac{K_{L}C_{e}}{1\;+\;K_{L}C_{e}}$			Freundlich Model qe=KFCe1n			Henry Model $q_{e}=K_{d}C_{e}$	
Cd^2+^	*K_L_* (L/mg)	*q_m_* (mg/g)	*R* ^2^	*K_F_* (L/mg)	*n*	*R* ^2^	*K_d_*	*R* ^2^
Unaged PE	0.1272	0.5192	0.9942	0.0583	0.3185	0.9962	0.0161	0.8722
Aged PE	0.2195	0.5816	0.9914	0.1174	0.4049	0.9841	0.0252	0.7973
Unaged PVC	0.0466	0.5977	0.9994	0.0190	0.4566	0.9975	0.0172	0.9466
Aged PVC	0.2026	0.9804	0.9896	0.1548	0.9259	0.9934	0.0454	0.8022
Pb^2+^	*K_L_* (L/mg)	*q_m_* (mg/g)	*R* ^2^	*K_F_* (L/mg)	*n*	*R* ^2^	*K_d_*	*R* ^2^
Unaged PE	0.2343	0.2354	0.9709	0.0481	0.1887	0.9656	0.0086	0.8069
Aged PE	0.1993	0.3329	0.9945	0.0772	0.2740	0.9575	0.0147	0.7876
Unaged PVC	0.0904	0.3926	0.9901	0.0390	0.3247	0.9815	0.0138	0.8795
Aged PVC	0.1366	0.4559	0.9904	0.0776	0.3861	0.9656	0.0209	0.8126

## Data Availability

Data will be made available on request.

## Supplementary Materials

The following supporting information can be downloaded at https://www.mdpi.com/article/10.3390/molecules31183181/s1, Figure S1: Elemental spectra of MPs after adsorption; Table S1: Adsorption kinetics and isotherm models; Table S2: Elemental analysis table corresponding to the energy spectra of MPs after adsorption. Table S3. Fold changes in BET specific surface area and equilibrium Cd^2+^ and Pb^2+^ adsorption capacity after representative aging treatments.
