# Supplementary material for: Surface Oxidation and Defect Formation Under Five Photoaging Regimes Enhance Cd2+ and Pb2+ Adsorption by Polyethylene and Polyvinyl Chloride Microplastics
Source: Molecules. 2026 Sep 10;31(18):3181. doi: 10.3390/molecules31183181 (PMC13608925; doi:10.3390/molecules31183181)
Supplement: Supplementary file 1 [file molecules-31-03181-s001.zip › molecules-4495107-supplementary.pdf]

Table S1. Adsorption kinetics and isotherm models.

Model	Equation	Linear form
Pseudo-first-order kinetic model	$q_t = q_e(1 - e^{-K_1 t})$	$\ln(q_e - q_t) = \ln q_e - k_1 t$
Pseudo-second-order kinetic model	$q_t = \frac{q_e^2 k_2 t}{1 + q_e k_2 t}$	$\frac{t}{q_t} = \frac{1}{K_2 q_e^2} + \frac{1}{q_e} t$
Langmuir	$q_e = q_m \frac{K_L C_e}{1 + K_L C_e}$	$\frac{C_e}{q_e} = \frac{1}{K_l q_m} + \frac{C_e}{q_m}$
Freundlich	$q_e = K_F C_e^{1/n}$	$\ln q_e = \ln K_F + \frac{1}{n} \ln C_e$
Henry	$q_e = K_d C_e$	-

t: Time (min), k_1: Rate constant of the pseudo-first-order kinetic model (/min), k_2: Rate constant of the pseudo-second-order kinetic model (g/(mg·min)), b: Langmuir isotherm constant, q_m: Maximum monolayer adsorption capacity (mg/g), K_l: Langmuir isotherm constant (L/mg), K_F: Freundlich capacity coefficient (mg/g)·(L/mg)$^{1/n}$, n: Freundlich adsorption intensity, K_d: Linear distribution coefficient of the contaminant between the solid and liquid phases (L/g)

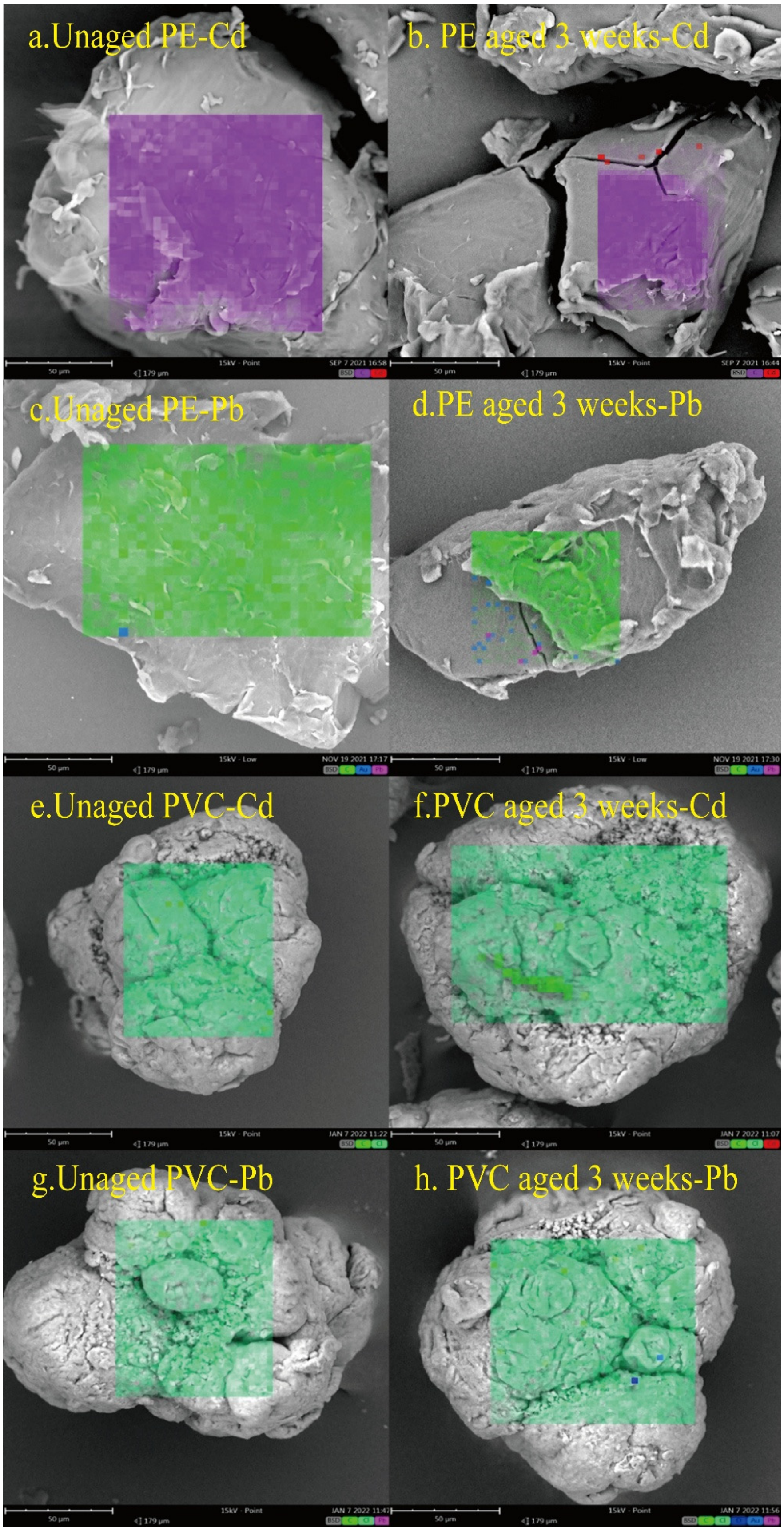

Figure S1. Elemental spectra of MPs after adsorption.

Table S2. Elemental analysis table corresponding to the energy spectra of MPs after adsorption.

Energy spectrum labeling	Element	Atomic percentage (%)	Weight percentage (%)
a	C	99.93	99.34
	Cd	0.07	0.66
b	C	99.83	98.44
	Cd	0.17	1.56
c	C	98.12	76.08
	Pb	0.09	1.15
d	C	98.03	75.19
	Pb	0.14	1.85
e	C	60.42	34.09
	Cl	39.58	65.91
f	C	57.41	31.03
	Cl	42.29	67.45
	Cd	0.30	1.52
g	C	56.78	30.64
	Cl	43.16	68.75
	Pb	0.07	0.61
h	C	41.81	23.03
	Cl	32.41	52.70
	O	25.14	18.45
	Pb	0.09	0.85

Table S3. Fold changes in BET specific surface area and equilibrium Cd^{2+} and Pb^{2+} adsorption capacity after representative aging treatments.

Polymer	Aged sample	BET surface area before aging ($m^2\ g^{-1}$)	BET surface area after aging ($m^2\ g^{-1}$)	BET ratio	Cd^{2+} adsorption before aging ($mg\ g^{-1}$)	Cd^{2+} adsorption after aging ($mg\ g^{-1}$)	Cd^{2+} ratio	Pb^{2+} adsorption before aging ($mg\ g^{-1}$)	Pb^{2+} adsorption after aging ($mg\ g^{-1}$)	Pb^{2+} ratio
PE	PE-UV/Cl, 3 weeks	0.1240	1.0787	8.70	0.2389	0.4508	1.89	0.1458	0.2641	1.81
PVC	PVC-UV/H_2O_2, 3 weeks	0.8112	1.1030	1.36	0.2443	0.8379	3.43	0.2175	0.3565	1.64
